# Improved contractile potential in detrusor microtissues from pediatric patients with end stage lower urinary tract dysfunction

**DOI:** 10.3389/fcell.2022.1007265

**Published:** 2022-10-04

**Authors:** Tim Gerwinn, Souzan Salemi, Larissa J. Schori, Dafni Planta, Daniel Eberli, Maya Horst

**Affiliations:** ^1^ Division of Pediatric Urology, University Children’s Hospital Zurich, Zurich, Switzerland; ^2^ Children’s Research Center, University Children’s Hospital Zurich, Zurich, Switzerland; ^3^ Laboratory for Urologic Oncology and Stem Cell Therapy, Department of Urology, University Hospital Zurich, Zurich, Switzerland

**Keywords:** bladder, pediatric, smooth muscle, neurogenic, lower urinary tract dysfunction, extracellular matrix, spheroid

## Abstract

Autologous cell-based tissue engineering has been proposed as a treatment option for end stage lower urinary tract dysfunction (ESLUTD). However, it is generally accepted that cells isolated from patient bladders retain the pathological properties of their tissue of origin and therefore need to be improved before they can serve as a cell source for tissue engineering applications. We hypothesize that human three-dimensional (3D) microtissues of detrusor smooth muscle cells (SMCs) are valuable *ex vivo* disease models and potent building blocks for bladder tissue engineering. Detrusor SMCs isolated from bladder wall biopsies of pediatric ESLUTD patients and healthy controls were expanded and cultured into 3D microtissues. Gene and protein analyses were performed to explore the effect of microtissue formation on SMC viability, contractile potential, bladder wall specific extracellular matrix (ECM) composition and mediators of ECM remodeling. Through microtissue formation, remodeling and intensified cell-cell interactions, the ESLUTD SMCs lost their characteristic disease phenotype. These microtissues exhibited similar patterns of smooth muscle related contractile proteins and essential bladder wall-specific ECM components as microtissues from healthy control subjects. Thus, the presented data suggest improved contractile potential and ECM composition in detrusor SMC microtissues from pediatric ESLUTD patients. These findings are of great relevance, as 3D detrusor SMC microtissues might be an appropriate cell source for autologous cell-based bladder tissue engineering.

## Introduction

End stage lower urinary tract dysfunction (ESLUTD) in children is mainly caused by congenital anomalies of the spinal cord (e.g., myelomeningocele, tethered cord) and bladder outlet obstruction in congenital posterior urethral valves (PUV). ESLUTD is characterized by a small bladder capacity, reduced bladder wall compliance and a consequently increased intravesical pressure (Gamé et al., 2010). If left untreated, or after exhaustion of conservative therapy, it can lead to kidney damage, reduced quality of life and increased mortality (Wishahi, 2021).

To date, there is no curative drug therapy, and surgery to control intravesical pressure by bladder augmentation using intestinal segments is merely symptomatic and comes with multiple short- and long-term complications (Hoen et al., 2017). Bladder tissue engineering (TE), currently discussed as a possible surgical alternative, has not been successful up to this point. As the pathophysiology of ESLUTD is not yet fully understood on a cellular level, basic research must gain better insights into the fundamental changes that lead to disease-typical alterations in order to ultimately offer improved therapies.

Previous analyses showed alteration of detrusor smooth muscle cells (SMCs), as well as fibrotic transformation of the bladder wall surrounding the detrusor. Important findings for SMC deterioration comprise a decreased contractile function of ESLUTD tissue compared to healthy controls (Johal et al., 2021a; Johal et al., 2021b). Unfortunately, SMCs seem to maintain this contractile impairment after isolation and cultivation *in vitro* (LIN et al., 2004). Several studies revealed changes in multiple genetic pathways involved in muscle development and muscle contraction (Dozmorov et al., 2007; Hipp et al., 2008), and detailed analysis of ESLUTD smooth muscle tissue showed significantly lower amounts, and altered composition of smooth muscle specific contractile protein (Eberli et al., 2018). Fibrotic bladder wall remodeling in ESLUTD by replacement of smooth muscle with extracellular matrix (ECM) has been reported in multiple studies (Eberli et al., 2018; Johal et al., 2021a; Johal et al., 2021b). The loss of smooth muscle is compensated by a buildup of collagens (DEVEAUD et al., 1998; Kaplan et al., 1997), contributing to bladder impairment by limiting contractility and bladder compliance (Fry et al., 2018). It is currently not established whether ESLUTD patient-derived cells are a suitable cell source for autologous TE applications.

*In vitro* characterization of SMCs derived from ESLUTD bladders has been performed exclusively in two-dimensional (2D) cell culture. However, cell cultures in 2D layers on plastic cell culture dishes are far from reproducing physiologic circumstances and therefore cannot represent complex *in vivo* conditions. During the last decade, literature has indicated that three-dimensional (3D) cell culture models free of foreign materials, can produce more physiologically relevant data (Fennema et al., 2013). The cells within 3D spheroids or microtissues are forced to interact exclusively with neighboring cells from their unique microenvironment, and form their own ECM, guiding their 3D assembly (Fennema et al., 2013). These intensified cell-cell interactions lead to a closer resemblance of *in vivo* gene expression and protein synthesis (Antoni et al., 2015). 3D cell culture thus seems to be a potent tool to better understand the underlying pathology on a cellular level. We recently reported on the successful generation of 3D spheroids from rat derived detrusor SMCs (Gerwinn et al., 2021).

With this study, we intended to generate human detrusor SMC microtissues derived from pediatric healthy controls and pediatric ESLUTD patients. To our knowledge, this is a novel approach, as 3D microtissues have never been described to study changes in human ESLUTD detrusor SMCs (Vasyutin et al., 2019).

We hypothesize, that 3D detrusor SMC microtissues are valuable *ex vivo* disease models for future drug treatments applications, and potent cellular building blocks for future bladder TE projects.

## Materials and methods

### Patients and ethics

Bladder wall biopsies of pediatric ESLUTD patients were taken during bladder augmentation at the University Children’s Hospital Zurich between February 2020 and September 2021. Healthy control tissue biopsies were taken from pediatric patients undergoing ureteric re-implantation for treatment of vesicoureteral reflux (VUR) without any signs for ESLUTD in their personal history, pre-operative ultrasounds or voiding cystourethrogram in the same period. This study was reviewed and approved by the cantonal ethics committee of Zurich (BASEC 2016-01287). The patients/participants and or their legal guardians provided their written informed consent to participate in this study.

### Bladder wall biopsies, human smooth muscle cell isolation and culture

Full thickness biopsies were taken from the anterior aspect of the bladder wall. The urothelial layer was removed. Detrusor samples were transferred to the laboratory in sterile lactated ringer solution on ice. Bladder biopsies were washed in PBS +3% Penicillin/Streptomycin/Fungizone for 10 min. Tissue was minced, and digested using collagenase type 1-S 0.2% (C1639, Sigma, St. Louis, Missouri, United States) and dispase 0.4% (Gibco, Grand Island, New York, United States). SMCs were cultured in medium containing DMEM/F12 + GlutaMAX (Invitrogen, Waltham, Massachusetts, United States), 10% fetal bovine serum (FBS; Merck, Darmstadt, Germany), 1% Penicillin/Streptomycin, 0.5 ng/ml human basic fibroblast growth factor (hbFGF; Sigma, St. Louis, Missouri, United States), 5 ng/ml human epidermal growth factor (hEGF; Sigma, St. Louis, Missouri, United States), and 5 μg/ml human insulin (Sigma, St. Louis, Missouri, United States). Culture dishes were incubated at 37°C in a humidified atmosphere with 5% CO_2_. Medium was changed every 3 days and SMCs were used up to passage 5.

### Smooth muscle cells microtissue production

Sphericalplate 5D^®^ (Kugelmeiers, Zurich, Switzerland) was used for SMC microtissue production according to established protocols (Gerwinn et al., 2021). The Sphericalplate 5D^®^ is a 24 well plate of which 12 wells have a micro structured well bottom with 750 non-adhesive micro cavities per well, where SMCs gather to form the microtissues. The SMCs were seeded in 2 ml of culture medium with a calculated 1500SMCs/microtissue. Following our previous experience (Gerwinn et al., 2021), microtissues were harvested and used for analysis on the 2nd day of culture.

### Live–dead dual staining study

Microtissues were stained with Calcein-AM and propidium iodide (PI) dual staining to ensure viability. Calcein AM is used to evaluate cell viability, as it is only converted into its green-fluorescent form, Calcein, by active metabolism within a living cell. PI is a fluorescent stain interacting with nucleic acids. A healthy, intact cell membrane would inhibit the cellular uptake of PI and therefore prevent staining of live cells. Briefly, 1 ml of fresh culture medium containing 1 μl Calcein-AM (10 μg/μl) (Invitrogen, Waltham, Massachusetts, United States), 1 μl PI (10 μg/μl) (Sigma, St. Louis, Missouri, United States), and 1 μl Hoechst (10 μg/μl) (Thermo Fisher, Waltham, Massachusetts, United States) was added to the microtissues. The samples were incubated at 37°C and 5% CO_2_ for half an hour. The supernatant was removed, and the microtissues were washed two times with PBS, and fixed with 4% paraformaldehyde (PFA) for 15 min. After two additional PBS washing steps the microtissues were transferred to a glass slide and DAKO mounting media was added. For PI positive control, microtissues were fixed in 4% PFA overnight at 4°C and stained in a similar way. Imaging was done using an inverted microscope (Leica THUNDER Imaging System, Wetzlar Germany).

### Total RNA isolation

Microtissues were lysed in TRI Reagent Solution (AM9738, Thermo Fisher, Waltham, Massachusetts, United States) for total RNA isolation according to manufacturer’s protocol. Briefly, 500 μl Tri Reagent was added, to lyse the samples. Chloroform (0.2 ml per 1 ml Tri Reagent) was added to the homogenate, and vigorously vortexed for 15 s. After incubation for 3 min at room temperature, the mixture was centrifuged at 14’000 rpm at 4°C for 15 min, separating it into three phases. The upper colorless phase was transferred to a fresh Eppendorf tube and equivalent volume of isopropanol was added. Following, 1 μl of GlycoBlue Coprecipitant (Invitrogen, Waltham, Massachusetts, United States) was added. The mixture was kept at −80°C, overnight. The next day, samples were centrifuged at 14’000 rpm at 4°C for 60 min to pellet RNA. After removal of the supernatant two washing steps with 500 μl 70% ethanol were performed. Residual ethanol was evaporated at 37°C for 5 min in a heating block with open tube cap. RNA was dissolved in 50 μl double-distilled and RNAse free water (ddH2O) by passing the solution through a pipette tip a few times. The solution was heated to 65°C for 10 min and put on ice immediately afterwards. RNA was measured using NanoDrop (Thermo Scientific, Waltham, Massachusetts, United States). RNA samples were stored at −80°C until use.

### Quantitative real-time PCR

1 μg of total RNA was reverse-transcribed to cDNA with random primers (High-Capacity cDNA reverse transcription, Applied Biosystems, Waltham, Massachusetts, United States). The samples (15 ng of cDNA per sample) were analyzed by measuring the cycle threshold (CT) values, which were between 17 and 35 in all samples. We evaluated the SMC specific contractile marker genes for α-smooth muscle actin (α-SMA), calponin, smoothelin and myosin heavy chain 11 (MYH11), the bladder wall specific extracellular matrix components collagen I, collagen III, elastin and fibronectin and the collagen cleaving matrix metalloproteinases (MMP) MMP1, MMP2, and MMP14 and their inhibitory regulators TIMP1 and TIMP2 as well as transforming growth factor beta 1 (TGF-beta 1). Gene expression was quantified relative to the housekeeping gene GAPDH. Details of primers used are provided in Table 1.

**TABLE 1:** qRT-PCR primers TaqMan Gene Expression Assay (FAM).

Gene ID (Name)	Assay ID	Company
ACTA2 (α-smooth muscle actin)	Hs05005341_m1	Thermo Fisher
CNN1 (calponin)	Hs00154543_m1	
SMTN (smoothelin)	Rn01453095_m1	
MYH11 (myosin heavy chain 11)	Hs00224610_m1	
GAPDH (Glyceraldehyde 3-phosphate Dehydrogenase)	Hs99999905_m1	
COL1A1 (collagen I)	Hs00164004_m1	
COL3A1 (collagen III)	Hs00943809_m1	
FN1 (fibronectin)	Hs01549976_m1	
ELN (elastin)	HS00355783_m1	
MMP1 (matrix metalloproteinase-1)	Hs00899658_m1	
MMP2 (matrix metalloproteinase-2)	Hs01548727_m1	
MMP14 (matrix metalloproteinase-14)	Hs00237119_m1	
TGF-beta 1 (transforming growth factor beta 1)	Hs00998133_m1	
TIMP1 (tissue inhibitor of metalloproteinases 1)	Hs01092511_m1	
TIMP2 (issue inhibitor of metalloproteinases 2)	Hs00234278_m1	

### Immunofluorescent staining

Microtissues were fixed with 4% PFA for 15 min at room temperature, permeabilized for 15 min with 0.5% triton X-100 in PBS at room temperature and blocked with 5% bovine serum albumin (BSA) in PBS +1% goat-serum + 0.2% triton for 1 h at room temperature. Samples were stained at 4°C overnight using the following primary antibodies for the contractile SMC proteins: α-SMA, calponin, smoothelin and MYH11. The slides were incubated with a Cy3-conjugated secondary antibody at room temperature for 1 h.

For the ECM proteins, collagen I, collagen III, elastin and fibronectin were used. The slides were incubated with a FITC-conjugated secondary antibody at room temperature for 1 h. Unbound primary and secondary antibodies were removed with 2 × 5 min washing in PBS after their respective incubation periods. Counterstaining of the nuclei was done with DAPI. Images were taken with an inverted microscope (Leica THUNDER Imaging System, Wetzlar, Germany). For negative controls, the primary antibody was omitted. Detailed antibody information is given in Table 2.

**TABLE 2:** Antibodies Immunofluorescence.

Antibodies	Company	Catalog number	Assays and dilution used
Anti-α-Smooth Muscle Actin antibody, Mouse monoclonal	Sigma-Aldrich	A5228	IF (1 : 200)
Anti-α-Smooth Muscle Actin antibody, Mouse monoclonal	Novus	NBP2-33006	WES (1 : 100)
Anti-Smoothelin antibody, Rabbit polyclonal	Novus	NBP2-37931	IF (1 : 20)
			WES (1 : 100)
Anti-Calponin antibody, Mouse monoclonal	Sigma-Aldrich	C2687	IF (1 : 100)
			WES (1 : 200)
Anti-Myosin heavy chain 11 antibody, mouse monoclonal	Santa Cruz	SC-6956	IF (1 : 5)
			WES (1 : 10)
Anti-Myosin heavy chain 11 antibody, mouse monoclonal	Novus	NBP2-44533	WES (1 : 50)
Anti-Collagen 1 antibody, rabbit monoclonal	Abcam	ab260043	IF (1 : 150)
Anti-Collagen 3 antibody, rabbit monoclonal	Abcam	ab7778	IF (1 : 150)
Anti-Elastin antibody, Mouse monoclonal	Abcam	ab9519	IF (1 : 100)
Anti-Fibronectin antibody, Mouse monoclonal	Santa Cruz	SC-59826	IF (1 : 300)
Anti-MMP1 antibody, mouse monoclonal	Novus	MAB901	WES (1 : 50)
Anti-MMP2 antibody, mouse monoclonal	Novus	NB200-114SS	WES (1 : 50)
Anti-MMP14 antibody, mouse monoclonal	Novus	MAB9181	WES (1 : 50)
Anti-TIMP1 antibody, goat monoclonal	R&D Systems	AF970	WES (1 : 100)
Anti-TIMP2 antibody, mouse monoclonal	Novus	MAB971	WES (1 : 50)
Anti-TGF-beta 1 antibody, rabbit monoclonal	Abcam	ab215715	WES (1 : 100)
DAPI (4′,6-diamidino-2-phenylindole)	Sigma-Aldrich	D9542	IF (1 : 400)
Rabbit CY3-conjugated secondary antibody	Sigma-Aldrich	AP132C	IF (1 : 500)
Mouse CY3-conjugated secondary antibody	Sigma-Aldrich	AP124C	IF (1 : 500)
Anti-Mouse FITC secondary antibody	BD Biosciences	555988	IF (1 : 500)
Anti-Rabbit FITC secondary antibody	Vector Laboratories	FI-1000–1.5	IF (1 : 500)
Anti-GAPDH antibody, mouse monoclonal	Novus	NB300-221	WES (1 : 100)

### Total protein isolation

Microtissues were harvested into an Eppendorf tube and washed in PBS. Western blot lysis buffer (50 mM Tris pH 7.4, 150 mM NaCl, 10% Glycerol, 1% Triton100, 2 mM EDTA, 10 mM NapyroP, 50 mM NAF, 200 uM Na3VO4, ddH2O) was added to lyse the samples. Samples were vortexed thoroughly and microtissues were disintegrated by needling. Samples were centrifuged for 20 min at 4°C with 14’000 rpm, the protein containing supernatant was transferred to a fresh tube. Total protein was measured with the BCA Protein Assay Kit (Thermo Scientific, Waltham, Massachusetts, United States).

### Immunoblotting

Protein concentration of 1.2—2.0 mg/ml was used for the automated western immunoblotting (Protein Simple WES, San Jose, California, United States) sample preparation, using the 12—230 kDa cartridge kit. The simple western assays are based on capillary electrophoresis, where denatured proteins are separated by molecular weight, immobilized by UV-light in the capillary, and directly detected within the capillary by primary and secondary antibodies. Primary antibodies for α-SMA, calponin, smoothelin and MYH11, MMP1, MMP2, MMP14, TIMP1, TIMP2, TGF-beta 1 were used. The protein amount was normalized to GAPDH (internal control), quantified and analyzed using compass software (Protein Simple, San Jose, California, United States). For detailed antibody information and dilution see Table 2.

### Statistics

All experiments were performed in triplicate. Data are presented as mean ± standard error of the mean (SEM). Statistics was done using GraphPad Prism 9 (GraphPad Software, La Jolla, California, United States) by unpaired *t*-test. Statistical significance was defined as *p < 0.05.

## Results

### Patients

ESLUTD tissue was obtained from four pediatric patients (3 females, 1 male) (2x meningomyelocele, 1x caudal regression syndrome, 1x PUV) undergoing augmentation cystoplasty. Control tissue was taken from five female pediatric patients undergoing ureteric re-implantation for treatment of VUR. The mean age for ESLUTD and control patients was 10^3/12^ (6^6/12^—13) years, and 3^4/12^ (1^1/12^—7^4/12^) years respectively. This represents a significant age difference between the two groups (*p = 0.02).

### Effect of microtissue formation on smooth muscle cell viability

**FIGURE 1:**
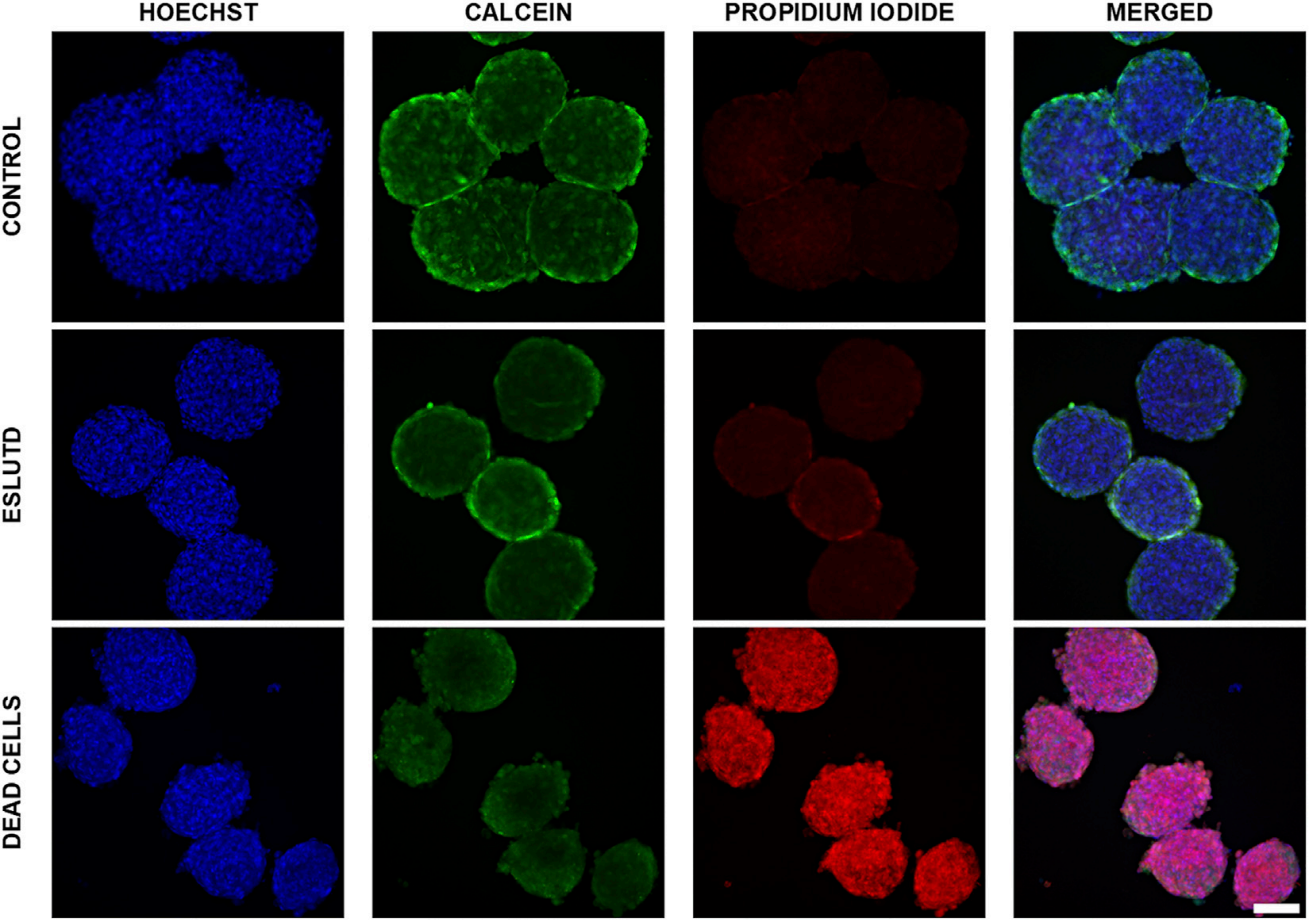
Live—dead dual staining of control and ESLUTD microtissues. Representative images for controls, ESLUTD and dead cells as reference for PI positivity. Pronounced Calcein positivity, macroscopic features and negligible PI uptake for control and ESLUTD microtissues indicate cell viability. Scale bar 100 μm.

Control and ESLUTD microtissues were clearly viable, as indicated by their healthy appearance with smooth surface and strong green Calcein coloration. This is supported by negligible red-fluorescent PI positivity, which is mostly overlap or auto-fluorescence of the microtissues **(** Figure 1 **)**.

Microtissues showed the typical Calcein staining distribution with higher intensity on the surface compared to the core area. This is indicating a proliferative surface zone, a quiescent intermediate layer and a necrotic core.

The dead cells show pronounced PI-positivity and cell membrane blebbing, a typical feature of cells undergoing apoptosis **(** Figure 1 **)**.

### Contractile potential of smooth muscle cell microtissues

**FIGURE 2:**
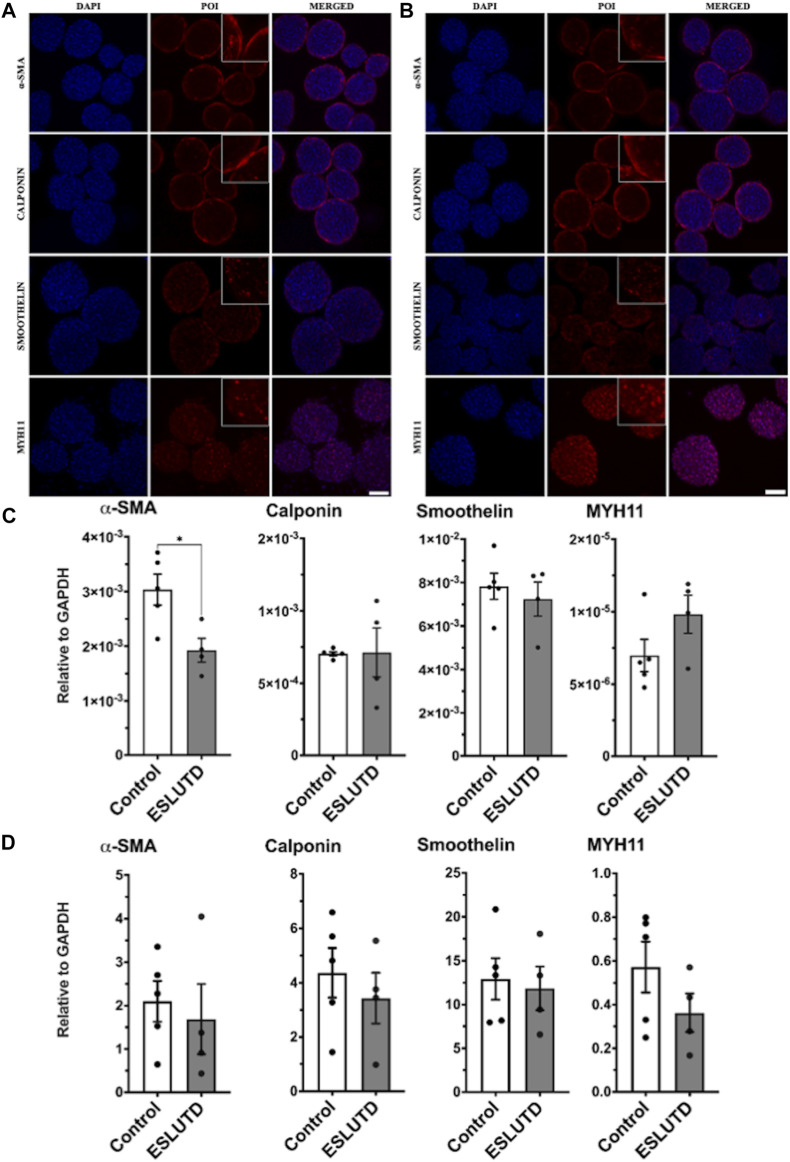
Smooth muscle specific contractile protein. **(A)** Immunofluorescence staining study for control and **(B)** ESLUTD microtissues shows expression of all examined SMC contractile marker proteins α-SMA, calponin, smoothelin and MYH11. Scale bar: 100 μm; zoomed images for more detail. **(C)** qRT-PCR gene expression of SMC specific markers displays significantly higher levels for α-SMA in controls compared to ESLUTD microtissues (*p < 0.05) while other SMC specific marker genes show no significant changes in ESLUTD compared to control. **(D)** Simple western immunoblotting of SMC contractile marker proteins shows that microtissues of control SMCs display a trend towards larger amounts of all SMC specific contractile proteins compared to ESLUTD, without reaching statistical significance. POI: protein of interest; α-SMA: alpha smooth muscle actin; MYH11: myosin heavy chain 11; SMC: smooth muscle cell; ESLUTD: end stage lower urinary tract dysfunction.

Gene expression patterns for smooth muscle specific contractile genes were analyzed **(** Figure 2C **)**. The statistical evaluation showed that the expression of α-SMA is significantly higher in healthy controls compared to ESLUTD with a mean of 0.0030 ± SEM 0.00028 for controls, compared to 0.0019 ± 0.00022 for ESLUTD (*p* = 0.02*). Calponin (0.00070 ± 1.4e-005; 0.00071 ± 0.00017) and smoothelin (0.0078 ± 0.00060; 0.0072 ± 0.00078) were expressed equally, whereas MYH11 (7.0e-006 ± 1.1e-006; 9.8e-006 ± 1.3e-006) showed a tendency towards higher gene expression in ESLUTD, considering a low overall induction, and without reaching statistical significance.

Morphological differences and changes in SMC specific contractile marker proteins were assessed with immunofluorescent staining for each patient. In line with the previously presented live - dead staining, the microtissues displayed a smooth and sphere like silhouette without signs for disturbance of the microtissues integrity **(** Figure 2 A,B **)**. Contractile protein distribution within the microtissues showed similar results for both groups. While α-SMA and calponin displayed a more peripheral or surface area staining, the distribution of smoothelin and MYH11 seemed to extend over the entire microtissue, including the intermediate and core zone. Both groups showed comparable staining intensities for the contractile proteins studied.

To further confirm our results, quantification of SMC specific contractile proteins was carried out by automated western immunoblotting. For both, controls as well as ESLUTD, all investigated proteins could be detected **(** Figure 2D **)**. Direct comparison of the two groups showed a tendency towards larger amounts of α-SMA (control 2.1 ± 0.47; ESLUTD 1.7 ± 0.81), calponin (4.4 ± 0.91; 3.4 ± 0.94), smoothelin (13 ± 2.4; 12 ± 2.5) as well as MYH11 (0.57 ± 0.12; 0.36 ± 0.088) in healthy controls. Nevertheless, the data do not show significant differences between healthy and ESLUTD microtissues. There were accentuated differences between individual patients. See Supplementary Figure S1 for representative bands.

### Bladder wall specific extracellular matrix composition in microtissues

**FIGURE 3:**
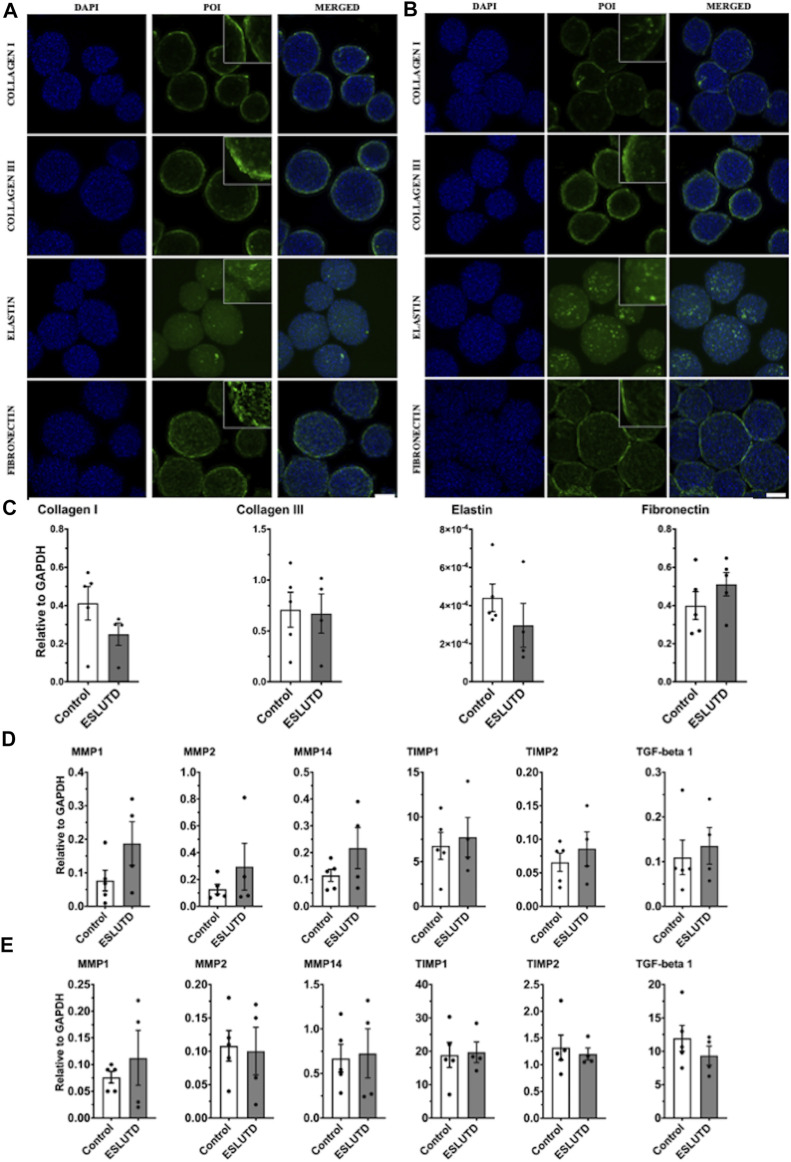
Bladder wall specific extracellular matrix and remodeling. **(A)** Immunofluorescence staining for control and **(B)** ESLUTD microtissues show similar expression for bladder wall specific ECM marker proteins collagen I, collagen III, elastin and fibronectin. Scale bar: 100 μm; zoomed images for more detail. **(C)** qRT-PCR gene expression of bladder wall specific ECM components revealed no statistically significant difference. However, all ECM genes except fibronectin seem to be more strongly represented in controls. **(D)** qRT-PCR of ECM remodeling mediators consistently show higher gene expression in ESLUTD microtissues. **(E)** Simple western immunoblotting of ECM remodeling mediators showed lower amounts of MMPs, while TIMPs and TGF-beta 1 were clearly more present than GAPDH. No significant differences could be identified. POI: protein of interest; MMP: matrix metalloproteinase; TIMP: tissue inhibitor of matrix metalloproteinase; TGF-beta 1: transforming growth factor-beta. ESLUTD: end stage lower urinary tract dysfuntion.

Gene expression analysis of bladder wall specific ECM did not reveal any significant changes **(** Figure 3C **)**. Expression of collagen I (control 0.41 ± 0.088; ESLUTD 0.25 ± 0.059), collagen III (0.71 ± 0.17; 0.67 ± 0.19) and elastin (0.00044 ± 7.3e-005; 0.00030 ± 0.00011) tended to be higher in controls, while fibronectin (0.40 ± 0.073; 0.51 ± 0.061) was more abundant in ESLUTD. The collagen III: I ratio was higher in ESLUTD microtissues 2.57 ± 0.30, albeit not reaching statistical significance when compared to control 1.82 ± 0.23.

ECM protein expression pattern and staining intensity within the microtissues was evaluated for the predominant components of the human bladder wall, collagen I, collagen III, elastin and fibronectin **(** Figure 3A,B **)**. The distribution of all investigated proteins showed the same pattern with respect to surface to core spreading when comparing the two groups. Collagen I and III appear closer to the microtissues surface with lower network formation towards its core, while fibronectin developed a widespread and dense network all over the microtissue. Distribution of elastin is also widespread but less intense, indicating lower levels of the protein of interest (POI).

### Extracellular matrix remodeling in microtissues

Quantification of gene expression for enzymes and cytokines involved in ECM remodeling did not reveal significant differences between controls and ESLUTD **(** Figure 3D **),** although the expression of all examined genes was higher in ESLUTD patients.

We analyzed the collagen cleaving matrix metalloproteinases (MMPs) MMP1 (control 0.077 ± 0.031; ESLUTD 0.19 ± 0.065), MMP2 (0.13 ± 0.036; 0.23 ± 0.18) and MMP14 (0.12 ± 0.023; 0.22 ± 0.077), the endogenous tissue inhibitors of matrix metalloproteinases (TIMPs) TIMP1 (6.76 ± 1.51; 7.73 ± 2.21) and TIMP2 (0.066 ± 0.014; 0.086 ± 0.025) and the multifunctional and profibrotic cytokine TGF-beta 1 (0.11 ± 0.039; 0.14 ± 0.041).

To support our results, MMPs, TIMPs and TGF-beta 1 were quantified on the protein level. Protein concentration in general was low for MMP1 (control 0.076 ± 0.012; ESLUTD 0.11 ± 0.051), MMP2 (0.11 ± 0.023; 0.10 ± 0.036) and MMP14 (0.67 ± 0.16; 0.73 ± 0.28). TIMP1 (18.84 ± 3.78; 19.70 ± 3.06) was extensively, and TIMP2 (1.32 ± 0.23; 1.20 ± 0.11) clearly represented in both groups. TGF-beta 1 (11.94 ± 1.94; 9.35 ± 1.42) was found in abundance in relation to GAPDH **(** Figure 3E **)**. See Supplementary Figure S1 for representative bands.

## Discussion

We hypothesized, that human 3D detrusor SMC microtissues are valuable *ex vivo* disease models, and potent building blocks for bladder TE projects. Most importantly, we found that both human control and ESLUTD detrusor SMCs formed 3D microtissues. Through microtissue formation, remodeling and intensified cell-cell interactions, the ESLUTD SMCs lost their characteristic disease phenotype. ESLUTD SMCs showed a pattern of smooth muscle related contractile proteins and essential bladder wall specific ECM components similar to healthy controls.

Simulation of *in vivo* cell physiology is key in basic research. 2D cell culture is mostly used to characterize disease and to identify treatment targets *in vitro*. However, the 2D environment is far from the conditions *in vivo* and has been brought into question in the last decade. We therefore aimed to harness 3D cell cultures, which mimic organ specific cell behavior *ex vivo* and open up new opportunities for disease modelling and testing of novel therapeutics (Fatehullah et al., 2016). 3D cultured cell aggregates possess a greater potential to re-create *in vivo* microenvironment with cell-cell interactions in every direction. Cells independently secrete the ECM in which they are embedded, and exclusively interact with cells from their microenvironment of origin (Fennema et al., 2013). Thus, 3D cell culture display patterns of gene expression and protein synthesis that are closer to *in vivo* conditions, leading to more realistic genotypes and phenotypes (Antoni et al., 2015). 3D cell culture has already been used in basic urological research to investigate urinary tract infections (UTIs) and bladder cancer (Vasyutin et al., 2019). The use of animal models in biomedical research is increasingly becoming an ethical, political and financial issue. 3D cell culture gained attention in the context of the 3R-principle as it may bridge the gap between cell culture and animal work, allowing the examination of actual human cells (Fitzgerald et al., 2015).

The composition of contractile proteins in ESLUTD SMC microtissue implies an improvement of their contractile phenotype towards control. ESLUTD microtissues are indistinguishable from healthy controls in immunofluorescence. Quantification of contractile protein shows a subtle tendency towards larger amounts in control microtissues, yet not reaching statistical significance. The same applies to gene expression, except for a significantly higher expression of α-SMA in controls. The more specific SMC markers calponin, smoothelin and MYH11 were similarly expressed in both groups.

The SMC phenotype develops in a continuous transition between a normal contractile and a synthetic, more proliferative phenotype. The contractile phenotype is characterized by a contractile response to external stimuli and high expression levels of contractile proteins. The synthetic isoform presents with extensive ECM deposition and lower expression levels of contractile proteins (Beamish et al., 2010). Important markers for the contractile SMC phenotype are α-SMA, calponin, smoothelin and MYH11. α-SMA is the most commonly used marker for an SMC lineage. It has a low specificity for the contractile SMC phenotype, as it is expressed early on and is also detectable in skeletal muscle, myofibroblasts and endothelial cells (Beamish et al., 2010). Calponin and smoothelin are expressed later on and more specifically in contractile SMCs (Beamish et al., 2010). MYH11 is the most mature contractile protein in SMC differentiation, and was proposed as the most definite marker for contractile functional SMCs (Beamish et al., 2010). The distinction between the different isoforms of MYH is of importance, as MYH10 (nonmuscle myosin heavy chain B) is rather a marker for a synthetic SMC phenotype. MYH10 is predominantly expressed in embryonic SMCs and its expression is re-induced in vascular injury or atherosclerotic blood vessels (Beamish et al., 2010). A switch between contractile and synthetic SMC phenotype and *vice versa* appears possible; the underlying mechanisms are not yet understood (Beamish et al., 2010). It is however of great importance, as SMCs isolated from diseased bladders retain their pathological properties in 2D cell culture, and perhaps are not suitable for autologous cell-based TE. Staining of neurogenic lower urinary tract dysfunction (NLUTD) bladder tissue revealed loss of detrusor smooth muscle density, in line with reduced expression of calponin and smoothelin and corresponding qRT-PCR alterations for calponin, smoothelin and MYH11 (Eberli et al., 2018). The corresponding NLUTD SMCs, isolated in 2D cell culture, showed significantly less expression of smoothelin and MYH11 compared to normal bladder SMCs (Eberli et al., 2018). This was recently reconfirmed by our group where SMCs isolated from the very same pediatric ESLUTD patients mentioned above in paragraph 2.1. Showed significantly lower amounts of contractile protein markers in conventional 2D cell culture than SMCs from control patients (Salemi S. et al., unpublished data). An attempt to determine whether SMCs isolated from neuropathic bladders possess and retain functional differences in 2D cell culture exposed different characteristics compared to healthy control (LIN et al., 2004). Markedly increased cell proliferation, a decreased adhesion capacity, and significantly decreased contractile potential *in vitro* contractile assays (51% less than normal in serum-free condition *p = 0.05), attributed to changed SMC contractile apparatus, of NLUTD SMCs were verified (LIN et al., 2004). It was concluded that the possibility of using autologous neuropathic SMCs for TE requires further investigation. In line with our results, no differences in α-SMA and MYH western blotting were detected. However, neither the molecular weight nor the investigated isoform of MYH was disclosed (LIN et al., 2004). Quantification of α-SMA, calponin, smoothelin and MYH11 by immunoblotting, as presented here, allows a better representation of the continuum of SMC differentiation (Beamish et al., 2010). To further examine the changes in 2D cell culture, a cDNA microarray analysis of more than 1000 genes revealed 17 potentially involved genes with an upregulated expression of at least two-fold in neuropathic bladders (Dozmorov et al., 2007). Previous observations in 2D cell culture could thus be reproduced on a gene level, reconfirming that cells from neuropathic bladders require improvement prior to their use for TE purposes (Dozmorov et al., 2007). More than 300 genes involved in key developmental processes, such as cell maturation, cell differentiation, contractile potential and ECM production, were identified as potential targets for the *in vitro* improvement of neurogenic SMCs derived from patients with myelomeningocele.

We could not detect significant differences in bladder wall specific ECM components by immunofluorescence staining and gene expression analysis. Collagen I and collagen III gene expression was higher in the control samples, which is neither consistent with our previous experience, nor with the composition of ESLUTD bladder tissue samples described in the literature. The frequently described shift to an altered collagen III: I ratio in ESLUTD tissue could be detected in the SMC microtissues studied here, but without reaching statistical significance.

The ECM network of the bladder wall is in a ceaseless cross-talk with its hosted cells, strongly influencing their proliferation, differentiation and homeostasis. Consideration of the ECM is crucial to understand pathophysiologic changes in bladder impairment. Fibrotic remodeling in ESLUTD bladders is characterized by an excessive buildup of connective tissue leading to stiffening and loss of compliance (Fry et al., 2018). *In vivo*, this not only impairs the filling phase of the bladder but also its contractile potential, as a more rigid ECM is a critical mediator for cell behavior and leads to long lasting alterations in SMCs (Aitken and Bägli, 2009). Two recent studies analyzed the mechanical differences between pediatric ESLUTD bladder biopsies and controls. The association of ECM buildup, stiffening of the bladder wall, and decreased contractility was evaluated. A combined population of 28 children with either NLUTD or PUV (14 congenital spinal cord defect, 14 PUV), sowed that diseased bladder tissue had a significantly reduced contractile response to carbachol and electric field stimulation, and a greater passive tissue stiffness in response to stretch (Johal et al., 2021a; Johal et al., 2021b). Tissue staining revealed a significant increase of connective tissue and compensatory loss of detrusor muscle (Johal et al., 2021a; Johal et al., 2021b). The similar results obtained in NLUTD and PUV tissue are comprehensible. Essentially, sub-vesical obstruction plays a central role in bladder wall changes in both diseases. PUVs prevent resistance free urine drainage by blocking the urethra. In high risk NLUTD, bladder outlet obstruction can occur during micturition by unsynchronized closure of the bladder sphincter owed to neurologic impairment (Tanaka et al., 2021). In depth analysis of collagen subtype composition in healthy bladder wall tissue showed a well-adjusted gene expression for all types of collagen, while neurogenic bladder tissue showed an unbalanced increase in the type III: type I collagen gene expression (DEVEAUD et al., 1998; Kaplan et al., 1997). The changes on the mRNA level were in line with a statistically significant increase in collagen type III: type I protein and an absolute increase in collagen III in neurogenic bladders compared to control (DEVEAUD et al., 1998). A higher percentage of collagen type III in collagen fibrils consisting of type III and type I collagen reduces their mechanical stiffness. This implies, that bladder wall compliance is not necessarily based on the total increase of collagens, but is also associated with the composition of collagen fiber bundles (Asgari et al., 2017). The bladder wall responds to extensive stretch by altering levels of MMPs, TIMPs and TGF-beta 1. This induces remodeling of the ECM composition and an increase in bladder wall stiffness. MMPs are a family of proteases that can degrade a variety of ECM components (Jackson et al., 2010). Their endogenous inhibitors TIMPs downregulate MMP activity; increased TIMP levels are therefore associated with fibrotic remodeling (Arpino et al., 2015). TGF-beta 1 is a multifunctional cytokine involved in proliferation, differentiation and wound healing in many tissues. It activates fibroblasts and SMCs and leads to increased deposition of ECM among others (Mann et al., 2001; Ong et al., 2021). Analysis of collagen cleaving MMPs, TIMPs and TGF-beta 1 in ESLUTD and control microtissues revealed similar results on both gene and protein levels. This is conceivable since the analysis of the ECM composition of the microtissues revealed no significant differences.

Our study shows that human detrusor derived SMC microtissues with improved contractile potential and ECM composition are promising building blocks for bladder TE. For the first time we can demonstrate here that human bladder SMCs behave differently when incorporated in microtissues. It seems that diseased bladder SMCs normalize their pathological phenotype at least partially in 3D cell culture.

Based on our collected data, the underlying mechanisms for the observed improved contractile phenotype cannot be explained. The anticipated improved contractile potential seen on the protein and gene level must be further confirmed by functional contractile *in vitro* assays in the future.

Among the multiple factors influencing the presented data is an immense interpatient diversity regarding disease severity, previous interventions or surgeries, as well as the exposure to multiple drugs administered for symptom control or treatment of UTIs. The significant age difference between ESLUTD patients and controls is unavoidable as open ureteral re-implantation is indicated in a younger patient collective than augmentation cystoplasty for ESLUTD. Furthermore, the number of evaluated patients is limited. Thus, detailed analysis at both the gene and protein level were used to validate our results. Therefore, our study cannot claim to present data representative for all ESLUTD patients.

In conclusion, our data implies an improved contractile potential and ECM composition in pediatric ESLUTD SMC microtissues. These findings are of great relevance, as 3D detrusor SMC microtissues might be an appropriate source for autologous cell-based bladder TE.

## Data Availability

The original contributions presented in the study are included in the article/Supplementary Material, further inquiries can be directed to the corresponding author.

## Supplementary material

Supplementary Figure S1 Representative bands for simple western immunoblotting.Molecular weight for GAPDH ≈ 40 kDa, α-SMA ≈ 43 kDa, calponin ≈ 34-45 kDa, smoothelin ≈ 130 kDa, MYH11 ≈ 200 kDa, MMP1 ≈ 54 kDa, MMP2 ≈ 45 kDa, MMP14 ≈ 65 kDa, TIMP1 ≈ 38 kDa, TIMP2 ≈ 30 kDa, TGF-b1 ≈ 58 kDa.

## References

[B1] Aitken K. J. Bägli D. J. (2009). The bladder extracellular matrix. Part I: Architecture, development and disease. Nat. Rev. Urol. 6 (11), 596–611. 10.1038/nrurol.2009.201 19890339

[B2] Antoni D. Burckel H. Josset E. Noel G. (2015). Three-dimensional cell culture: A breakthrough *in vivo* . Int. J. Mol. Sci. 16 (3), 5517–5527. 10.3390/ijms16035517 25768338 PMC4394490

[B3] Arpino V. Brock M. Gill S. E. (2015). The role of TIMPs in regulation of extracellular matrix proteolysis. Matrix Biol. 44-46, 247–254. 10.1016/j.matbio.2015.03.005 25805621

[B4] Asgari M. Latifi N. Heris H. K. Vali H. Mongeau L. (2017). *In vitro* fibrillogenesis of tropocollagen type III in collagen type I affects its relative fibrillar topology and mechanics. Sci. Rep. 7 (1), 1392. 10.1038/s41598-017-01476-y 28469139 PMC5431193

[B5] Beamish J. A. He P. Kottke-Marchant K. Marchant R. E. (2010). Molecular regulation of contractile smooth muscle cell phenotype: Implications for vascular tissue engineering. Tissue Eng. Part B Rev. 16 (5), 467–491. 10.1089/ten.TEB.2009.0630 20334504 PMC2943591

[B6] Deveaud C. M. Macarak E. J. Kucich U. Ewalt D. H. Abrams W. R. Howard P. S. (1998). Molecular analysis of collagens in bladder fibrosis. J. Urology 160 (4), 1518–1527. 10.1097/00005392-199810000-00108 9751406

[B7] Dozmorov M. G. Kropp B. P. Hurst R. E. Cheng E. Y. Lin H. K. (2007). Differentially expressed gene networks in cultured smooth muscle cells from normal and neuropathic bladder. J. Smooth Muscle Res. 43 (2), 55–72. 10.1540/jsmr.43.55 17598958

[B8] Eberli D. Horst M. Mortezavi A. Andersson K-E. Gobet R. Sulser T. (2018). Increased autophagy contributes to impaired smooth muscle function in neurogenic lower urinary tract dysfunction. Neurourol. Urodyn. 37 (8), 2414–2424. 10.1002/nau.23705 29797356

[B9] Fatehullah A. Tan S. H. Barker N. (2016). Organoids as an *in vitro* model of human development and disease. Nat. Cell Biol. 18 (3), 246–254. 10.1038/ncb3312 26911908

[B10] Fennema E. Rivron N. Rouwkema J. van Blitterswijk C. de Boer J. (2013). Spheroid culture as a tool for creating 3D complex tissues. Trends Biotechnol. 31 (2), 108–115. 10.1016/j.tibtech.2012.12.003 23336996

[B11] Fitzgerald K. A. Malhotra M. Curtin C. M. Fergal J. O. B. Caitriona M. O. D. (2015). Life in 3D is never flat: 3D models to optimise drug delivery. J. Control. Release 215, 39–54. 10.1016/j.jconrel.2015.07.020 26220617

[B12] Fry C. H. Kitney D. G. Paniker J. Drake M. J. Kanai A. Andersson K-E. (2018). Fibrosis and the bladder, implications for function ICI-RS 2017. Neurourol. Urodyn. 37 (4), S7–S12. 10.1002/nau.23725 30133788

[B13] Gamé X. Fowler C. J. Panicker J. N. (2010). Neuropathic bladder dysfunction. Trends Urol. Gynecol. Sex. Health 15 (1), 23–28. 10.1002/tre.133

[B14] Gerwinn T. Salemi S. Krattiger L. Eberli D. Horst M. (2021). Spheroids of bladder smooth muscle cells for bladder tissue engineering. Biomed. Res. Int. 2021, 9391575. 10.1155/2021/9391575 34805410 PMC8601859

[B15] Hipp J. A. Hipp J. D. Yoo J. J. Atala A. Andersson K. E. (2008). Microarray analysis of bladder smooth muscle from patients with myelomeningocele. BJU Int. 102 (6), 741–746. 10.1111/j.1464-410X.2008.07606.x 18336610

[B16] Hoen L. Ecclestone H. Blok B. F. M. Karsenty G. Phe V. Bossier R. (2017). Long-term effectiveness and complication rates of bladder augmentation in patients with neurogenic bladder dysfunction: A systematic review. Neurourol. Urodyn. 36 (7), 1685–1702. 10.1002/nau.23205 28169459

[B17] Jackson B. C. Nebert D. W. Vasiliou V. (2010). Update of human and mouse matrix metalloproteinase families. Hum. Genomics 4 (3), 194–201. 10.1186/1479-7364-4-3-194 20368140 PMC3525976

[B18] Johal N. Cao K. Arthurs C. Millar M. Thrasivoulou C. Ahmed A. (2021). Contractile function of detrusor smooth muscle from children with posterior urethral valves - the role of fibrosis. J. Pediatr. Urol. 17 (1), 100.e1–100100.e10. 10.1016/j.jpurol.2020.11.001 33214068 PMC9099076

[B19] Johal N. Cao K. X. Xie B. Millar M. Davda R. Ahmed A. (2021). Contractile and structural properties of detrusor from children with neurogenic lower urinary tract dysfunction. Biol. (Basel) 10 (9), 863. 10.3390/biology10090863 PMC8471516 34571740

[B20] Kaplan E. P. Richier J. C. Howard P. S. Ewalt D. H. Lin V. K. (1997). Type III collagen messenger RNA is modulated in non-compliant human bladder tissue. J. Urology 157 (6), 2366–2369. 10.1097/00005392-199706000-00115 9146672

[B21] Lin H-K. Cowan R. Moore P. Zhang Y. Yang Q. Peterson J. A. (2004). Characterization of neuropathic bladder smooth muscle cells in culture. J. Urol. 171 (3), 1348–1352. 10.1097/01.ju.0000108800.47594.8b 14767346

[B22] Mann B. K. Schmedlen R. H. West J. L. (2001). Tethered-TGF-β increases extracellular matrix production of vascular smooth muscle cells. Biomaterials 22 (5), 439–444. 10.1016/s0142-9612(00)00196-4 11214754

[B23] Ong C. H. Tham C. L. Harith H. H. Firdaus N. Israf D. A. (2021). TGF-β-induced fibrosis: A review on the underlying mechanism and potential therapeutic strategies. Eur. J. Pharmacol. 911, 174510. 10.1016/j.ejphar.2021.174510 34560077

[B24] Tanaka S. T. Yerkes E. B. Routh J. C. Tu D. D. Austin J. C. Wiener J. S. (2021). Urodynamic characteristics of neurogenic bladder in newborns with myelomeningocele and refinement of the definition of bladder hostility: Findings from the UMPIRE multi-center study. J. Pediatr. Urol. 17 (5), 726–732. 10.1016/j.jpurol.2021.04.019 34011486 PMC11008495

[B25] Vasyutin I. Zerihun L. Ivan C. Atala A. (2019). Bladder organoids and spheroids: Potential tools for normal and diseased tissue modelling. Anticancer Res. 39 (3), 1105–1118. 10.21873/anticanres.13219 30842139

[B26] Wishahi M. (2021). Lower urinary tract dysfunction in pediatrics progress to kidney disease in adolescents: Toward precision medicine in treatment. World J. Nephrol. 10 (4), 37–46. 10.5527/wjn.v10.i4.37 34430383 PMC8353602

