# Supplementary figures and images for: Improved contractile potential in detrusor microtissues from pediatric patients with end stage lower urinary tract dysfunction
Source: Front Cell Dev Biol. 2022 Oct 4;10:1007265. doi: 10.3389/fcell.2022.1007265 (PMC9577217; doi:10.3389/fcell.2022.1007265)

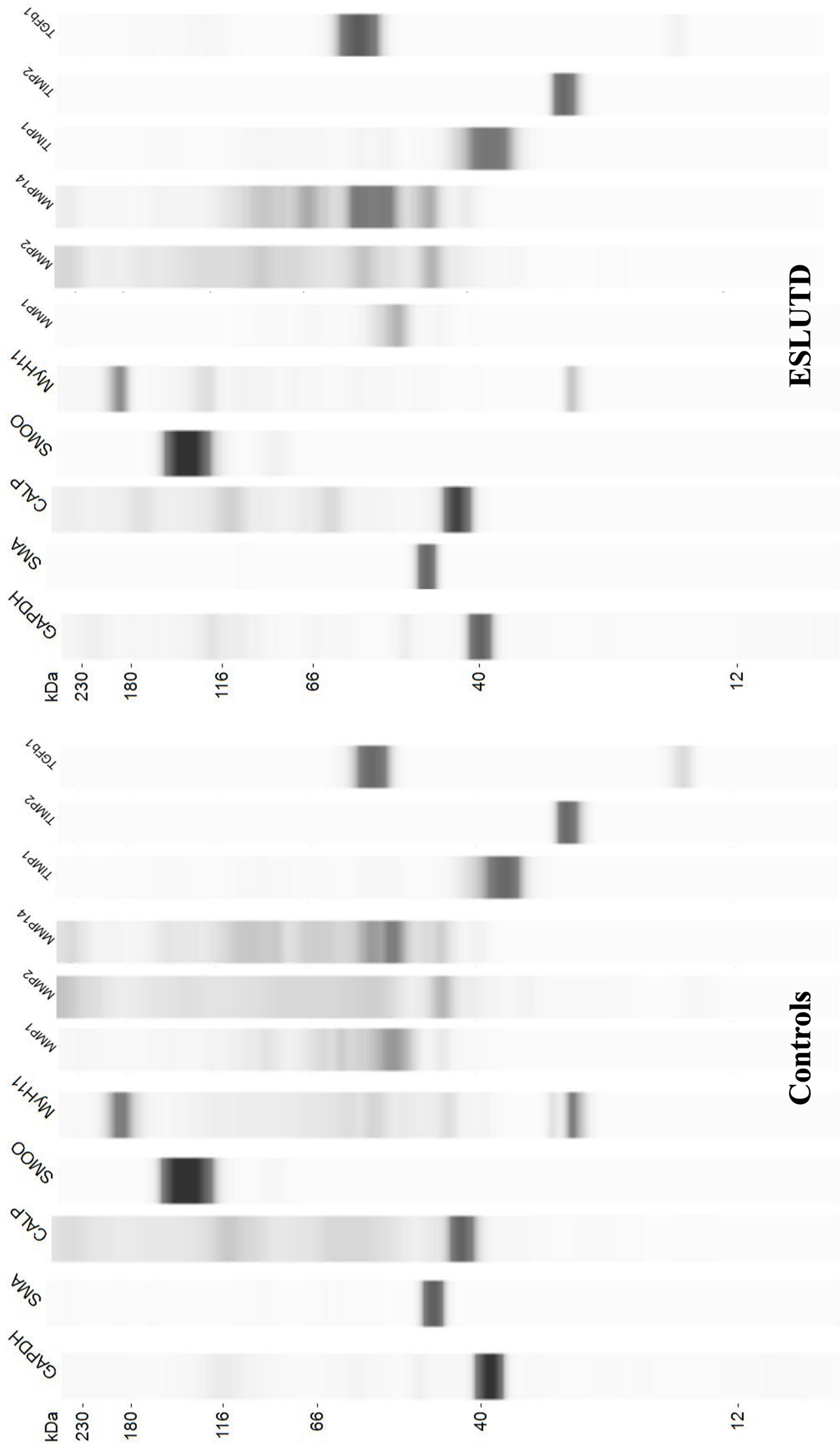

Supplement: Supplementary file 1 [file Image1.pdf]
